# Disease burden of myotonic dystrophy type 1

**DOI:** 10.1007/s00415-019-09228-w

**Published:** 2019-02-20

**Authors:** Erik Landfeldt, Nikoletta Nikolenko, Cecilia Jimenez-Moreno, Sarah Cumming, Darren G. Monckton, Grainne Gorman, Chris Turner, Hanns Lochmüller

**Affiliations:** 1 Institute of Environmental Medicine, Karolinska Institutet, Nobels väg 13, 17177 Stockholm, Sweden; 2 ICON plc, Stockholm, Sweden; 3 John Walton Muscular Dystrophy Research Centre, Institute of Genetic Medicine, Newcastle University, Newcastle upon Tyne, UK; 4 National Hospital for Neurology and Neurosurgery, Queen Square, University College London Hospitals NHS Foundation Trust, London, UK; 5 Institute of Molecular, Cell and Systems Biology, College of Medical, Veterinary and Life Sciences, University of Glasgow, Glasgow, UK; 6 Wellcome Trust Centre for Mitochondrial Research, Institute of Neuroscience, University of Newcastle, Newcastle upon Tyne, UK; 7 Department of Neuropediatrics and Muscle Disorders, Medical Centre, Faculty of Medicine, University of Freiburg, Freiburg, Germany; 8 Division of Neurology, Department of Medicine, Children’s Hospital of Eastern Ontario Research Institute, The Ottawa Hospital, University of Ottawa, Ottawa, Canada

**Keywords:** Disease burden, Quality of life, INQoL, DM1, Myotonic dystrophy, Genetics

## Abstract

**Objective:**

The objective of this cross-sectional, observational study was to investigate the disease burden of myotonic dystrophy type 1 (DM1), a disabling muscle disorder.

**Methods:**

Adults with DM1 were recruited as part of the PhenoDM1 study from Newcastle University (Newcastle upon Tyne, UK). Disease burden data were recorded through the Individualized Neuromuscular Quality of Life (INQoL) questionnaire. Results were examined by sex and clinical variables [e.g. the six-minute walk test (6MWT), the Mini Mental State Examination, and estimated progenitor and modal allele CTG repeat length].

**Results:**

Our sample consisted of 60 patients with DM1 (mean age: 45 years; 45% female). Muscle weakness and fatigue constituted the two most common disease manifestations, reported by 93% and 90% of patients, respectively, followed by muscle locking (73%). Most patients (> 55%) reported feeling anxious/worried, depressed, frustrated, and/or having low confidence/self-esteem, 23% and 33% indicated substantial impairment of daily and leisure activities, respectively, and 47% did not work as a consequence of the disease. Estimated progenitor CTG length corrected by age correlated surprisingly well with INQoL scores. Differences by sex were generally minor.

**Conclusion:**

We show that DM1 is associated with a substantial disease burden resulting in impairment across many different domains of patients’ lives, emphasizing the need for a holistic approach to medical management. Our results also show that the INQoL records relevant information about patients with DM1, but that further investigation of the psychometric properties of the scale is needed for meaningful interpretation of instrument scores.

## Introduction

Myotonic dystrophy type 1 (DM1) is a chronic, progressive, and disabling muscle disorder frequently involving other organ systems [1]. The disease is inherited in an autosomal dominant pattern, caused by expansion of a cytosine–thymine–guanine (CTG) triplet repeat in the *DMPK* gene, and represents the most common muscular dystrophy in adults with an estimated prevalence in Europe of between 10–18/100,000 [2]. Patients with DM1 typically experience muscle weakness and locking (i.e. myotonia), but the phenotypic spectrum is wide and symptoms are variable, which makes the identification and validation of suitable outcome measures for clinical research challenging [3].

The disease burden of DM1 has been investigated in several geographical settings using different study designs and rating-scales, including the Individualized Neuromuscular Quality of Life (INQoL), a patient-reported outcome (PRO) tool developed to measure the impact of neuromuscular disease on various aspects of patients’ life [4]. However, to date, no study has examined specific components of the disease burden of DM1 using the INQoL in a UK setting. The objective of this study was therefore to estimate the prevalence, severity, and difficulties of symptoms of DM1, the impact of the disease on daily, leisure, and work activities, patients’ independence and relationships, as well as emotional well-being and appearance as recorded through the INQoL in adult patients with DM1 from the UK.

## Materials and methods

### Study design and patient sample

The data reported as part of this work were collected in the Myotonic Dystrophy Type 1 Deep Phenotyping to Improve Delivery of Personalized Medicine and Assist in the Planning, Design and Recruitment of Clinical Trials (PhenoDM1) study (ClinicalTrials.gov identifier: NCT02831504). Patients with DM1 were identified and recruited from the specialist neuromuscular clinic at Newcastle University (Newcastle upon Tyne, UK). To be eligible to participate, patients were required to meet the following inclusion criteria: (i) ≥ 18 years of age, (ii) genetically confirmed diagnosis of DM1, (iii) able and willing to provide written informed consent to participate in the study, and (iv) ability to perform the 10 m walking test at selected pace without any assistance (walking devices allowed). Patients with congenital DM were not included in the study. To further minimize reporting bias, incomplete questionnaires or questionnaires with double answers were excluded. Study ethical approval was granted by the Newcastle and North Tyneside Ethics Committee (reference: NE/15/0178).

### Study procedures, outcomes, and genetic analysis

The disease burden data presented as part of this work were recorded directly from patients through the INQoL as part of their routine clinical follow-up. The INQoL encompasses a total of 45 questions covering the prevalence and impact of a set of common muscle disease symptoms [muscle weakness, muscle locking (i.e. myotonia), pain, fatigue, droopy eyelids (i.e. ptosis), double vision (i.e. diplopia), and swallowing difficulties (i.e. dysphagia)], the impact of muscle disease on daily, leisure, and work activities, patients’ independence, relationships, emotional well-being, and appearance (i.e. “looks”) [4]. In addition to descriptive analysis of the collected data, each item within the INQoL is scored on a seven-point Likert scale (ranging from 0 to 6, or 1 to 7), where a higher score represents a higher disease burden, and a total instrument score can also be calculated based on the outcomes of five subscales [4]. As part of the study procedures, we also recorded data from patients concerning their basic demographic and clinical characteristics, as well as results from the six-minute walk test (6MWT) and the Mini Mental State Examination (MMSE). The 6MWT was performed in a 25-m long corridor with input every minute following the agreed procedures at the third outcome measures in myotonic dystrophy type 1 (OMMYD-3) international workshop [3]. CTG repeat length was estimated from blood DNA by the small-pool PCR assay as described by Gomes-Pereira et al. [5] using the CTG repeat-flanking primers DM-C and DM-DR [6, 7]. Replicate reactions were separated by gel electrophoresis, Southern blotted and hybridised using a ^32^P-labelled 56 x CTG repeat probe. Bands were detected by autoradiography and sized by comparison against the DNA molecular weight marker, using CLIQS software (TotalLab UK Ltd.). The bottom edge of the expanded allele bands was used to determine the estimated progenitor allele length (ePAL), i.e. the number of CTG repeats inherited [7]. The densest part of the expanded allele bands was used to estimate the modal allele length at the time of DNA sampling.

### Statistical analysis

We calculated the distribution of replies [i.e. the proportion and a corresponding 95% confidence interval (CI)] across all items and levels within the INQoL. We also derived the proportion of patients at or above two specific level thresholds (i.e. the first level, and the three highest levels, respectively) to facilitate interpretation and investigate the proportion of patients with, e.g. no difficulties, and a considerable amount, very many, or an extreme amount of difficulties. In addition, we calculated mean scores for each item/domain, as well as the total score as described by Vincent et al. [4], for the total sample, and by sex. We compared the distribution of replies to INQoL items and instrument scores across strata using Welch *t* tests and Welch’s analysis-of-variance models. In addition, we estimated Pearson’s correlation coefficients to investigate the relationship between DM1 symptoms and amount and importance of difficulties, as well as INQoL scores, and CTG repeat length, 6MWT, and MMSE, respectively. Finally, to further explore the relationship between estimated progenitor CTG repeat length and INQoL scores, we fitted ordinary least squares regression models to the study data, with INQoL scores as dependent variables and estimated progenitor CTG repeat length and age, as well as an interaction variable between estimated progenitor CTG repeat length and age, as independent variables [7]. Estimated progenitor CTG repeat length was normalised by log transformation [7]. All analyses were conducted in Stata 14 or R.

## Results

The final sample consisted of *n* = 60 patients with DM1 who completed all sections of the INQoL in accordance with the instructions. These patients constituted a subset of the total PhenoDM1 study population (*n* = 110) who were invited, and subsequently agreed, to complete the INQoL as part of the study procedures (in addition to the clinical and genetic tests performed). Summary demographic and clinical characteristics of the study cohort are presented in Table 1. Age at baseline ranged between 18 and 77 years, age at first symptoms of DM1 between 0 and 65 years, 6MWT result between 10 and 693 meters, MMSE score between 16 and 30, estimated progenitor length between 58 and 592 CTG repeats, and modal allele length between 64 and 1290 CTG repeats. At baseline, approximately 15% of patients had pacemakers, 27% history of cataract surgery, and 13% received part-time ventilation support. In our sample, women with DM1 had fewer years of full-time education (13 vs. 15, *p* = 0.037) and were to a greater extent in a relationship or married (70% vs. 36%, *p* = 0.008) compared with men. No other significant differences in reported demographic or clinical characteristics were noted by sex.

**Table 1:** Demographic and clinical characteristics of the study sample

Age, mean (SD) years	45 (14)
Sex, female	27 (45%)
Age at first symptoms, mean (SD) years*	23 (16)
Part-time wheelchair dependency	9 (15%)
Six-minute walk test result, mean (SD) meters**	412 (151)
Mini Mental State Examination, mean (SD) score	29 (2)
Estimated progenitor CTG repeat length, mean (SD)*	239 (126)
Modal CTG repeat length, mean (SD)*	489 (267)
Education, mean (SD) years completed*	14 (3)
Married or in a relationship	29 (48%)
Current occupation	
Employed	15 (25%)
Retired	8 (13%)
Long-term sick leave	20 (33%)
Unemployed/other	17 (28%)

Data presented as *n* (%), if not specified otherwise. Total sample: *n* = 60, excluding missing values for *n* = 2 patients (*) and *n* = 1 patient (**)

### Prevalence, severity, and difficulties of symptoms of DM1

**Fig. 1:**
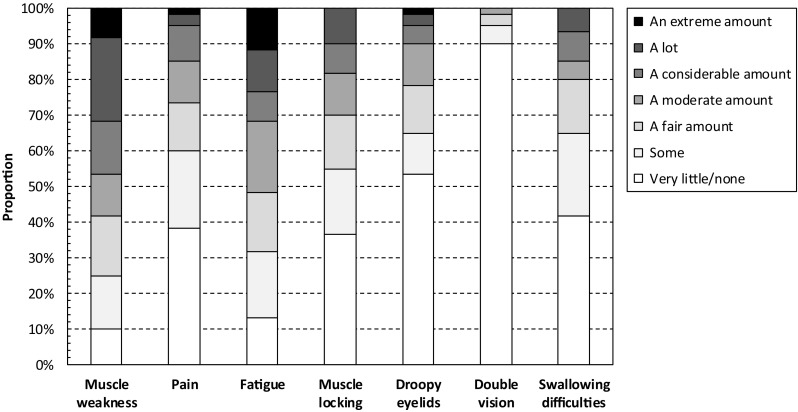
Prevalence of symptoms of DM1 as recorded using the INQoL. The Individualized Neuromuscular Quality of Life (INQoL) questionnaire

The INQoL records data on three aspects of common neuromuscular disease symptoms: (i) the prevalence and severity of symptoms, (ii) the amount of difficulties caused by the symptoms, and (iii) the importance of difficulties caused by symptoms. Prevalence of symptoms of DM1 as recorded using the INQoL is presented in Fig. 1. Muscle weakness and fatigue constituted the two most common disease manifestations, reported by 93% (95% CI 84–98%) and 90% (79–96%) of patients, respectively, followed by muscle locking (73%; 60–84%), swallowing difficulties (73%; 60–84%), pain (67%; 53–78%), droopy eyelids (60%; 46–72%), and double vision (18%; 9–30%). Moreover, 47% (34–60%) of patients stated that they experienced a considerable amount, a lot, or an extreme amount of muscle weakness. The corresponding estimates for fatigue were 32% (20–45%), muscle locking 18% (10–30%), swallowing difficulties 15% (7–27%), pain 15% (7–27%), droopy eyelids 10% (4–21%), and double vision 0% (0–6%), respectively.

**Fig. 2:**
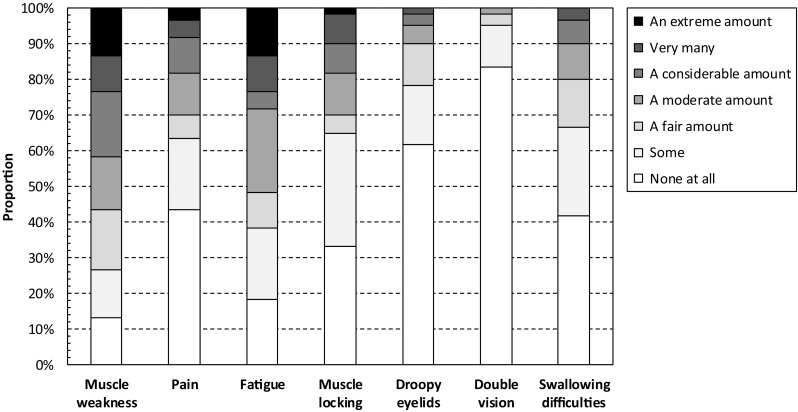
Amount of difficulties caused by symptoms as recorded using the INQoL. The Individualized Neuromuscular Quality of Life (INQoL) questionnaire

The amount of difficulties caused by symptoms as recorded using the INQoL is presented in Fig. 2. In total, 42% (29–55%) of patients reported that they experienced a considerable amount, very many or an extreme amount of difficulties with muscle weakness in their lives. The corresponding estimates for muscle locking were 18% (10–30%), pain 18% (10–30%), fatigue 28% (17–41%), droopy eyelids 5% (1–14%), double vision 2% (0–9%), and difficulties with swallowing 10% (4–21%), respectively.

**Fig. 3:**
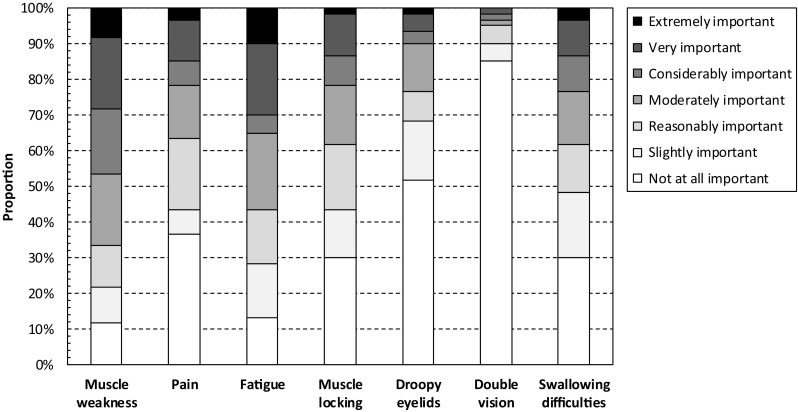
Importance of difficulties caused by symptoms as recorded using the INQoL. The Individualized Neuromuscular Quality of Life (INQoL) questionnaire

The importance to patients of difficulties caused by symptoms recorded using the INQoL is presented in Fig. 3. In total, 47% (34–60%) of patients reported that difficulties caused by muscle weakness were considerably, very, or extremely important to them. The corresponding estimates for muscle locking were 22% (12–34%), pain 22% (12–34%), fatigue 35% (23–48%), droopy eyelids 10% (4–21%), double vision 3% (0–12%), and difficulties with swallowing 23% (13–36%), respectively.

### Impact of DM1 on daily, leisure, and work activities

In total, 23% (13–36%) of patients reported that their ability to perform daily activities (e.g. washing, dressing, and housework) was considerably, very much, or extremely affected by DM1. The corresponding estimates for leisure activities were 33% (22–47%). Moreover, 62% (48–74%) of patients had no paid employment; 47% (34–60%) due to the disease. Among patients with paid employment, 13% (6–25%) reported that their work activities were considerably, very much, or extremely affected by DM1.

### Impact of DM1 on patients’ independence and relationships

Concerning help from other people in carrying out daily activities, in total, 27% (16–40%) of patients reported that they do not need any assistance. In contrast, 33% (22–47%) of patients reported that they need a considerable amount, very much, or an extreme amount of assistance, 32% (20–45%) rated their level of independence as quite bad, bad, or the worst it could possibly be, 48% (35–62%) stated that the lack of independence was considerably, very, or extremely important to them, and only 3% (0–12%) that their level of independence was exactly as they would like it to be.

**Fig. 4:**
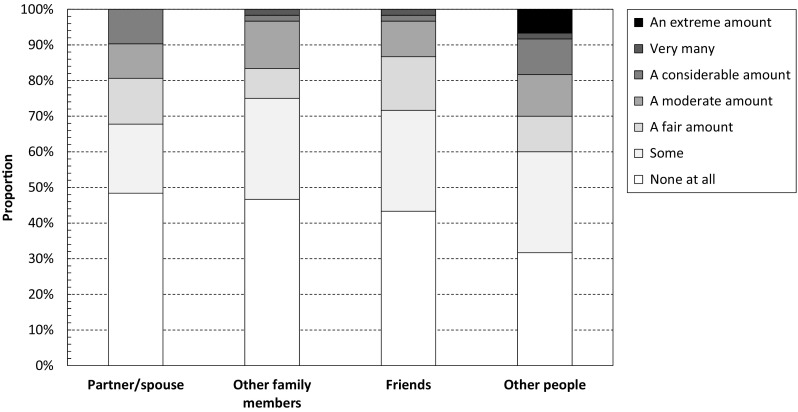
Relationship difficulties caused by DM1 as recorded using the INQoL. The Individualized Neuromuscular Quality of Life (INQoL) questionnaire

Figure 4 shows difficulties in relationships caused by DM1. In total, 52% (38–65%) of patients were in a relationship, and of them, 48% (30–67%) reported that their disease did not cause any difficulties for their partner/spouse, and 10% (2–26%) that it resulted in a considerable amount, very many, or an extreme amount of difficulties. The corresponding estimates for other family members were 47% (34–60%) and 3% (0–12%), friends 43% (31–57%) and 3% (0–12%), and other people (e.g. strangers, acquaintances, and colleagues) 32% (20–45%) and 18% (10–30%), respectively. Yet, across the four categories, only 8% (3–18%), 7% (2–16%), and 5% (1–14%), respectively, indicated that their close family friendships, close friendships, and relationships with other people were exactly as they would like.

### Impact of DM1 on emotional well-being and appearance

In total, 68% (55–80%) of patients reported feeling anxious/worried, and 15% (7–27%) to a substantial degree (i.e. considerably, very much, or extremely). The corresponding estimates for depressive feelings were 55% (42–68%) and 13% (6–25%), for frustration 68% (55–80%) and 30% (19–43%), and for low confidence/self-esteem 77% (64–87%) and 35% (23–48%), respectively. Concerning appearance, 18% (10–30%) of patients reported no impact of DM1 on their looks, and 25% (15–38%) that their looks were considerably, very much, or extremely affected by the disease. Yet, only 10% (4–21%) stated that appearance was important to them, and of these, 40% (28–53%) considerably, very, or extremely important.

### INQoL instrument scores

INQoL domain and total scores, where a higher score represents a higher disease burden, are presented in Table 2.

**Table 2:** INQoL subscale and total scores

	Total (*n* = 60)	Sex	
		Males (*n* = 33)	Females (*n* = 27)
Muscle weakness	53 (28)	55 (30)	52 (26)
Muscle locking	32 (28)	31 (29)	32 (26)
Pain	30 (29)	27 (27)	34 (31)
Fatigue	48 (29)	43 (27)	55 (31)
Activities	46 (29)	47 (31)	44 (28)
Independence	41 (30)	41 (34)	40 (25)
Relationships	26 (19)	27 (20)	23 (19)
Emotions	34 (25)	30 (22)	40 (27)
Body image	42 (27)	41 (28)	42 (26)
Total^a^	45 (22)	45 (23)	45 (21)

Data presented as mean (SD). A higher score resents a higher disease burden, and vice versa

^a^The total INQoL score was calculated as described by Vincent et al. [4]

### Relationship between DM1 symptoms and amount and importance of difficulties

The amount of muscle weakness, pain, fatigue, muscle locking, droopy eyelids, double vision, and swallowing difficulty, respectively, was all significantly associated with the amount of difficulties caused by each symptom (all *ρ* > 0.73, *p* < 0.001), and the amount of difficulties was associated with the importance of difficulties (all *ρ* > 0.76, *p* < 0.001).

### Relationship between sex and disease burden of DM1

We identified no significant differences between women and men with DM1 concerning the crude overall prevalence of symptoms (all *p* > 0.189), but found that a larger proportion of women with DM1 experienced a considerable amount, lot or, an extreme amount of fatigue (48% vs. 18%, *p* = 0.015) and depressive feelings (26% vs. 3%, *p* = 0.017). In addition, there was a trend that a larger proportion of women rated the importance of difficulties associated with fatigue as considerably, very, or extremely (48% vs. 24%, *p* = 0.059). Concerning impact on daily life, a larger proportion of men (59% vs. 30%, *p* = 0.025) reported that their muscle condition caused difficulties in their relationships with their friends, and more men than women rated their level of independence as quite bad, bad, or the worst it could possibly be (42% vs. 19%, *p* = 0.044). No other significant differences in INQoL outcomes and scores were identified in our analyses by sex.

### Relationship between clinical variables and INQoL scores

Results from our correlation analysis showed that estimated progenitor CTG repeat length was significantly associated with muscle weakness score (*ρ* = 0.38, *p* = 0.003) and independence score (*ρ* = 0.34, *p* = 0.008), modal CTG repeat length with muscle weakness score (*ρ* = 0.44, *p* < 0.001) and independence score (*ρ* = 0.35, *p* = 0.008), MMSE with INQoL independence score (*ρ* = − 0.30, *p* = 0.021), and 6MWT with muscle weakness score (*ρ* = − 0.64, *p* < 0.001), pain score (*ρ* = − 0.38, *p* = 0.003), activities score (*ρ* = − 0.59, *p* < 0.001), independence score (*ρ* = − 0.53, *p* < 0.001), body image score (ρ = -0.30, p = 0.023), and total score (*ρ* = − 0.46, *p* < 0.001). No other significant differences were identified in our correlation analysis of clinical variables and INQoL scores. Finally, outcomes from our regression analysis showed that age and estimated progenitor CTG repeat length were able to explain a non-trivial proportion of the variance in several INQoL domains: muscle weakness score (*R*^2^ = 0.21, *p* = 0.001), fatigue score (*R*^2^ = 0.09, *p* = 0.043), muscle locking score (*R*^2^ = 0.090, *p* = 0.038), activities score (*R*^2^ = 0.18, *p* = 0.003), independence score (*R*^2^ = 0.23, *p* < 0.001), relationship score (*R*^2^ = 0.10, *p* = 0.036), emotions score (*R*^2^ = 0.12, *p* = 0.021), and total score (*R*^2^ = 0.19, *p* = 0.002).

## Discussion

To the best of our knowledge, this study represents the first comprehensive examination of the disease burden of DM1 in the UK as recorded though the INQoL. Our results show that the vast majority of affected patients suffer from some degree of muscle weakness, muscle locking, pain, fatigue, and difficulties with swallowing, and that these symptoms collectively have a non-trivial impact on their ability to perform activities of daily living. Indeed, in our sample, 23% of patients indicated that their leisure activities were considerably, very much, or extremely affected by DM1, 47% did not work due to their illness, 33% reported requiring a considerable amount, very much, or an extreme amount of assistance, and 52% stated that their illness caused difficulties in their relationship with their partner/spouse. Our data also show that DM1 is often associated with impairment in psychological well-being, including anxiety, depressive feelings, frustration, and low confidence/self-esteem. Taken together, these findings underscore the detrimental impact of DM1 on affected patients and highlight the substantial unmet medical need in this disease population.

In line with expectations and previous research [8–11], our INQoL results show that muscle weakness and fatigue constitute two of the most common manifestations of DM1, affecting the vast majority of patients. In addition, these symptoms were also found to be associated with the most difficulties to the patients. We identified no significant differences concerning the crude prevalence of muscle weakness by sex, but did find, in contrast to previous research [12] that a significantly larger proportion of women (48% vs. 18%) experienced a considerable amount, a lot, or an extreme amount of fatigue.

Considering emotional well-being in DM1, most patients in our sample reported feeling anxious/worried, depressed, and/or frustrated to some degree, and many also indicated low confidence/self-esteem. Data indicating impaired mental health in DM1 have been previously reported [8–10, 13–16]. Interestingly, in the present study, we found evidence suggesting that women with DM1 were much more likely to report depressive feelings than men, a result also noted in previous research [8]. Yet, as discussed by Peric et al. [15], it is not known if these psychological traits constitute symptoms or manifestations of DM1 (i.e. as part of the underlying pathological process), or if they are developed as a consequence of living with the disease, or a combination of both. Nonetheless, given the prevalence observed in our study, mental health in patients with DM1 warrants further exploration.

Previous studies [8–10, 17] have estimated mean INQoL muscle weakness scores at between 49 and 70, muscle locking 38 and 63, pain 25 and 42, fatigue 42 and 60, activities 35 and 55, independence 30 and 47, social relationships 10 and 23, emotions 24 and 44, body image 6 and 62, and total score 19 and 52. Our estimates for the total sample all lie within these ranges, with the exception of social relationships [26], indicating a relatively higher burden on this aspect of life in our cohort. However, given the substantial variability in estimates (also between patients within samples), and considering non-trivial differences with respect to demographic and clinical characteristics of patients across studies, it is likely not meaningful to further compare our results. This is also related to the psychometric properties of the INQoL, where the Likert scoring algorithm would be expected to fail to adhere to the criteria of fundamental measurement, which is a requirement for invariant comparison [18, 19]. Put differently, although it is clear that a lower/higher score indicates a lower/higher disease burden, it is not clear how a specific score, or differences in scores at various points across the scale continuum should be interpreted. Indeed, the high association between DM1 symptoms and amount and importance of difficulties noted as part of this study suggest that combining Likert scores for these items may not be appropriate due to non-trivial item dependency. The ordinal nature of the INQoL is also likely to affect the possibility to detect and interpret changes in total scores over time. For example, despite worsening Muscular Impairment Rating Scale (MIRS) outcomes, Peric et al. [17] found that total INQoL scores, as well as several subscale scores, improved over the course of 6 years. Moreover, in a recent multicentre, single-blind, randomised trial, Okkersen et al. [20] identified no significant changes in total INQoL scores at 10 months from baseline between patients with DM1 receiving cognitive behavioural therapy plus standard care and optional graded exercise and those receiving standard of care alone, despite significant changes in the main endpoint (i.e. the DM1 activity and participation scale for clinical use [DM1-Activ^C^]) and other secondary endpoints. Additionally, Hamilton et al. [21] recently reported some inverse correlations between objective measures of disease severity and patient self-reported outcomes, suggesting disease-dependent lack of insight can sometimes mask physical symptoms.

The data presented as part of this study indicate that male patients may experience greater impairment in their relationships life compared to their female counterparts. Specifically, a significantly larger proportion of men (70% vs. 40%) reported of difficulties with their friends due to DM1, and men with the disease were much less likely to be married or in a relationship (36% vs. 70%). Evidence of a relatively larger impact on the social life of male patients has been reported for DM1 [22], as well as other chronic, disabling diseases, such as multiple sclerosis [23], and warrants further investigation.

Previous research [24–26] have demonstrated an association between CTG repeat length and age at onset in DM1. Such age at onset associations have been further improved by estimating the progenitor CTG allele length to overcome the confounding effects of age-dependent somatic expansion [7]. Previous attempts to correlate CTG length to progressive phenotypes have typically yielded poorer associations. Indeed, Antonini et al. [13] and Rakocevic-Stojanovic et al. [10] found no association between quality of life (as measured through the SF-36 and INQoL, respectively) and CTG repeat length. Here, we have estimated the progenitor CTG allele length to overcome the confounding effects of age-dependent somatic expansion and included an age-CTG interaction to account for the progressive nature of the symptoms and the inherent sampling biases found within DM1 families (due to the profound anticipation observed in DM1, older sampled individuals inherited smaller alleles, and vice versa). These analyses have yielded good correlations for overall INQoL score (*R*^2^ = 0.19) and for a number of the sub-domains: muscle weakness (*R*^2^ = 0.21), fatigue (*R*^2^ = 0.09), muscle locking (*R*^2^ = 0.09), activities (*R*^2^ = 0.18), independence (*R*^2^ = 0.23), relationships (*R*^2^ = 0.10) and emotions (*R*^2^ = 0.12). These data highlight the utility of careful genetic and statistical analyses that addresses the sampling biases inherent within DM1 populations. Outcome measures that are predicted by underlying genetic factors more likely to reflect primary disease processes and thus are more likely to respond positively to effective therapeutic interventions. Conversely, the lack of association with genotype for the pain, muscle locking, drooping, double vision, and body image scores suggests that these domains are not sensitive reporters of the disease process. Genotype to phenotype correlations such as these may therefore be of utility in selecting the most informative outcome measures for patient management and clinical trials.

Previous studies have also identified unexpected distributions of INQoL scores across measures of disease severity and age. For example, Peric et al. [17, 27] found that worsening of muscular weakness from mild to severe was associated with a lower disease burden (i.e. higher quality of life). In addition to the sampling biases discussed above, potential reasons for these counterintuitive findings include lack of disease awareness, in particular in patients with less severe disease [8], coping mechanism [28] (which has been identified for other rare, neuromuscular diseases, such as Duchenne muscular dystrophy [29]), and the properties of the scale, as discussed above.

Our study is subject to a few limitations. First, similar to most research of patients with rare diseases, our results are limited by a relatively small sample, which affects the precision and potentially also generalizability of derived estimates. This would be expected to be of particular relevance considering the heterogeneous presentation of DM1. Additionally, given the underlying variability in outcomes of interest and the magnitude of expected differences, conducting research in small patient populations may also have a negative impact on possibilities to perform meaningful evaluation of results across subgroups. For this reason, outcomes of our testing of differences by, for example, sex should be interpreted with some caution. Second, our self-reported data may be subjected to bias due to, e.g. incorrect reporting. Third, due to the observational nature of our data, we were unable to draw conclusions regarding causality. Concerning interpretation of results, it is also worth pointing out that we were unable to adjust our estimates for patients’ intelligence quotient (a variable that may have an impact on how patients perceive, and are able to report of, their disease burden), and that none of the patients in our sample received cognitive behavioural or exercise therapy (which recently have proven beneficial for the capacity for activity and social participation in DM1 [20]).

## Conclusions

We show that DM1 is associated with a substantial disease burden resulting in impairment across many different domains of patients’ lives, emphasizing the need for a holistic approach to disease management. Our results also show that the INQoL records relevant information about patients with DM1, but that further investigation of the psychometric properties of the scale is needed for meaningful interpretation of instrument scores.
